# A protocol to determine the least cost supply of coal to China with an installation-level optimization model

**DOI:** 10.1016/j.xpro.2022.101873

**Published:** 2022-11-23

**Authors:** Jorrit Gosens, Alex B.H. Turnbull

**Affiliations:** 1 Crawford School of Public Policy, Australian National University, Acton, ACT 2600, Australia; 2 Keshik Capital, Singapore 048583, Singapore

**Keywords:** Energy, Computer sciences

## Abstract

This protocol provides instructions on how to run a linear optimization model that determines the cost-optimal supply of coal, from Chinese and foreign mines, to satisfy a given demand for coal in Chinese power and steel plants. We describe how to download the code and required software and detail steps to insert third-party data. We further provide instructions to adjust and define the scenario settings, build and solve the optimization problem file, and visualize the model outcomes.

For complete details on the use and execution of this protocol, please refer to Gosens et al. (2022).^1^

## Highlights

• Protocol describes a model to determine the least cost supply of coal to China
• Describes how to download all required code and software and insert coal mine data
• Provides instructions to adjust scenario settings and run the optimization model
• Has instructions to expand node-and-link model and scripts to visualize outcomes

## Abstract (editor highlights)

Publisher’s note: Undertaking any experimental protocol requires adherence to local institutional guidelines for laboratory safety and ethics.

## Graphical abstract

**Figure:**
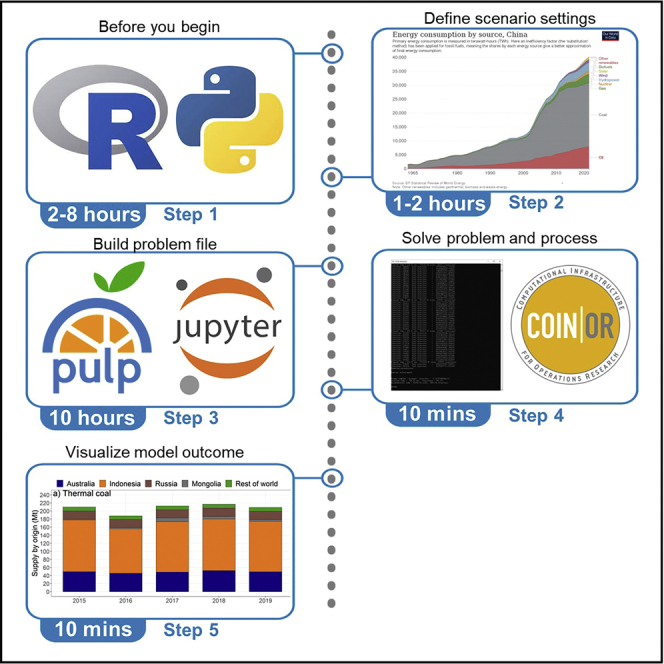

## Before you begin

This protocol provides instructions on how to run the “Installation-Level China Coal Model (IL-CCM)”,^2^ a linear optimization model that determines the cost-optimal supply of thermal and coking coal to Chinese power plants and steel plants. This model was used in a study that assessed how China’s decarbonization and energy security plans are set to reduce its future seaborne coal imports.^1^

Understanding the Chinese market is of key relevance to understanding international trade in coal. China is the largest importer of both thermal coal (used for power generation) and coking coal (used for steelmaking), being responsible for roughly a quarter of total global imports of both types of coal.^3^^,^^4^ Further, China itself produces vast quantities of coal itself. Over 2019–2021, a little over 50 percent of global coal production came from Chinese mines.^4^ China’s future coal imports are subject to two pressures. First, China has announced a net-zero emission target for 2060 with an interim target of peaking carbon emissions by 2030, and this will inevitably involve reduced consumption of coal.^5^^,^^6^ Second, China has been seeking to improve its energy security, or the share of energy derived from domestic sources, by opening up additional coal mining capacity, and improving the transport infrastructure to get this coal to its domestic power and steel plants.^7^

Determining the likely volumes of coal shipped from any mine, or from any country, to the Chinese market, requires understanding the relative competitiveness between these suppliers. A common approach to modelling such relative competitiveness is with so-called “multiregional coal supply models”, with previous applications to global coal markets,^8^^,^^9^^,^^10^^,^^11^ and a long list of China-focused coal market analyses.^7^^,^^12^^,^^13^^,^^14^^,^^15^^,^^16^^,^^17^^,^^18^

The regions, or nodes, in such models can either produce or consume coal (or both), and are connected with other nodes through links, which represent transport infrastructure, and allow one node to supply coal demand in another node. These models include information on coal production capacity and cost for each producing node (in coal mines), transport capacity and cost for each link (railways, roads, waterways, and trans-shipment points), and coal demand levels in consuming nodes.

In comparison to these previous models, the IL-CCM stands out because of its very high geo-spatial resolution. The most detailed previous analysis with this modelling method represented China’s coal production and demand conflated into provincial-level nodes, and the transport system conflated into inter-provincial links, for a total of 31 nodes and about 150 links.^7^ The IL-CCM has installation-level location and technical detail for each individual coal mine, power and steel plant, as well as for all connecting rail, highway, UHV transmission, and shipping networks, for a total of 12,161 nodes and 40,396 links (for more detail see “Methods S1”). A backcasting exercise shows that this high level of detail greatly helps model accuracy. The model predicts levels of Chinese coal imports from different suppliers that very closely match historically observed levels (Figure 1).

**Figure 1:**
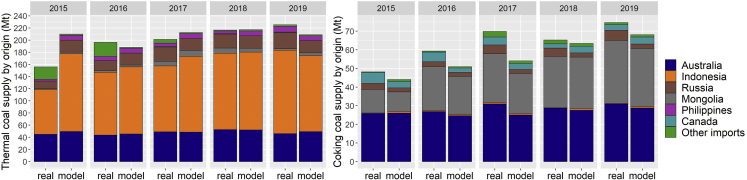
IL-CCM model prediction accuracy in backcasting exercise: Historical (real) and predicted (model) levels of Chinese thermal and coking coal imports, by origin, for the years 2015–2019

The IL-CCM model uses input data stored in spreadsheet files. Data preparation, processing of the solution file created by the optimization software, and visualization of results is done in R. The file with the linear problem written out as a set of mathematical expressions (a .lp file), for use in linear optimization, is created in Jupyter on a cloud computing instance, whilst solving the problem file is done locally with the CPLEX solver.

We provide all code and data used in this protocol in a public repository, whereas earlier work with similar methods, at best, provided only mathematical descriptions of the optimization model,^7^^,^^8^ or in one exception, model descriptions and data,^10^ and is the first Star Protocol reporting a process for an energy system optimization model. This repository further includes scripts for editing the networks, and local and freeware alternatives for the optimization steps. The user will need a dataset on mine-level production costs and capacity. This is commercial data that could not be provided with the public repository. For further model details including a full mathematical description, see “Methods S1”.

### Download IL-CCM data and code repository

**Timing: 10 min**

The IL-CCM repository includes all the required code and data to run the model. When editing files in this model, only change the values of input data, and do not change the names of files, or sheet or column names in spreadsheet files.

1. Download the repository with fully functional code, data, and folder structure from Zenodo: https://doi.org/10.5281/zenodo.7222764.
2. Extract the repository to any location on your computer.

***Note:*** Most of the scripts used below use relative file paths. Any absolute file reference in the below will use “∼/China-coal-model-star-protocol/” to indicate the location of the extracted repository on your machine.

### Get third-party coal mine data

**Timing: 1–2 h**

The repository described in the previous step does not include mine-level production costs, production capacity, and coal qualities, as this is proprietary third party data. The user can either use the same third-party data as was originally used in creating this model, or alternatively they may use their own coal mine data, or from any other commercial supplier, although it is substantially more work to insert such data into the model.

3. Purchase or prepare mine-level coal-mine data.
  a. Option 1: Purchase the “China and Mongolia coal supply” and the “Seaborne Export Thermal Curve” and “Seaborne Export Metallurgical Curve” from Wood Mackenzie (https://www.woodmac.com/).
  b. Option 2: Use any other data supplier or own assumptions. Data requirements are:
    i. A unique mine name for each mine.
    ii. Locational data: what province the mine (cluster) is in for Chinese mines, or what country a mine (cluster) is in for foreign mines.
    iii. A technical production capacity and total cash cost in US$/t.
    iv. Coal quality data, specifically: whether it is thermal, hard coking, semi-soft coking, or pulverized coal for injection, as well as calorific value (in kcal/t) for thermal coals.

***Note:*** the repository described in the step “Download IL-CCM data and code repository” still includes the coal mine data sheets with the required layout. These files have been censored by replacing data with arbitrarily picked numbers for key technical and economic indicators. That means the model will run fine, but will produce unrealistic results until these coal mine data sheets have been updated with real data.

### Install the required software

**Timing: 1–3 h**

4. Install R and associated software.
  ***Note:*** the versions mentioned in the steps below are the versions as used during the creation of this protocol. Older or newer versions may work as well.
  a. Download and install R version 4.0.5.
  b. Download and install R Tools version 4.0.
  c. Option 1 (Recommended): Install required packages using the environment file provided with the data and code repository.
    i. Open the file “China-coal-model-star-protocol.Rproj” in the data and code repository.
    ii. Install renv by typing:
      >install.packages('renv')
    iii. Install all required packages by typing:
      >renv::restore()
    iv. Type “y” when prompted “Do you want to proceed?”
  d. Option 2 (Alternatively): Install required packages manually.
    i. Install devtools by typing:
      >install.packages('devtools')
    ii. Install all required packages by typing:
      > require(devtools)
      > install_version("here", version = "1.0.1")
      > install_version("readr", version = "2.0.2")
      > install_version("readxl", version = "1.3.1")
      > install_version("tidyverse", version = "1.3.0")
      > install_version("stringr", version = "1.4.0")
      > install_version("formattable", version = "0.2.1")
      > install_version("ggplot2", version = "3.3.6")
      > install_version("ggpubr", version = "0.4.0")
      > install_version("RColorBrewer", version = "1.1.2")
      > install_version("scales", version = "1.1.1")
      > install_version("geosphere", version = "1.5.14")
      > install_version("ggmap", version = "3.0.0")
      > install_version("ggspatial", version = "1.1.6")
      > install_version("sf", version = "1.0.7")
      > install_version("rgeos", version = "0.5.9")
      > install_version("maps", version = "3.4.0")
      > install_version("rnaturalearth", version = "0.1.0")
      > install_version("rnaturalearthdata", version = "0.1.0")
5. Install Python and associated software.
  a. Download and install Anaconda 3 v2022.05.
  b. Open a Anaconda prompt (for instructions see: https://docs.anaconda.com/anaconda/install/verify-install/).
  c. Option 1 (Recommended): Install required software using the environment file provided with the data and code repository, by typing:
    > conda env create -f "∼ /China-coal-model-star-protocol/environment.yml"
  d. Option 2 (Alternatively): Install required software manually, by typing:
    > conda create -n china-coal-imports python=3.6.10 pandas=1.1.1 numpy=1.19.1 xlrd=1.2.0 spyder=4.1.5
    > conda activate china-coal-imports
    > conda install -c conda-forge pulp=2.3
6. Install the IBM ILOG CPLEX Optimization Studio (“CPLEX”).
  a. Browse to the CPLEX website (https://www.ibm.com/products/ilog-cplex-optimization-studio) and click “get the no-cost academic edition”.
  b. Register an account with your university email address.
  c. Download and install the CPLEX Optimization Studio.

### Sign up for and set up a cloud computing account

**Timing: 1–2 h**

7. Option 1: sign up with MS Azure.
  a. Browse to https://azure.microsoft.com/ and click “get started”.
  b. Sign up with your details and pick a subscription plan.
    ***Note:*** A free account will be sufficient for several model runs.
  c. Browse to https://portal.azure.com/#home and click “create a resource”.
  d. Search for “Azure Machine Learning” in the resources search box, click “create”.
  e. Choose configuration options (new resource group, new storage account, and workspace name; not relevant to the functioning of this code), and click “review and create”.
8. Option 2: sign up with AWS Sagemaker.
  a. Browse to https://aws.amazon.com/sagemaker/ and click “Get started with SageMaker”.
  b. Sign up with your details and login.
  c. Click on “get started” and then “set up sagemaker domain”.
  d. Choose configuration options (domain name; not relevant to the functioning of this code), and click “create”.
  e. Click on “Services”, then “Support”, then “create a case”, “Looking for service limit increases”.
  f. Select limit type “Sagemaker studio” form the dropdown, pick a region, and ask for a service limit increase to 1 for an instance (more info at: https://docs.aws.amazon.com/sagemaker/latest/dg/notebooks-available-instance-types.html).

***Note:*** We recommend using a “compute optimized” instance, with at least 16 virtual CPU and 32 GB of memory, i.e., an instance of type “ml.c5.4xlarge” or better.

***Note:*** this step is optional but recommended. The cloud computing service is used to run the code that builds the problem file for use in the optimization studio. This can be done on a desktop computer but will take considerable time. Below are instructions for two alternative popular providers of these cloud computing services.

### Sign up for a geocoding api

**Timing: 1–2 h**

9. Sign up for a Google Maps account.
  a. Browse to https://developers.google.com/maps and click “get started”.
  b. Sign up with your personal details.
  c. Browse to https://console.cloud.google.com/projectcreate and create a new project.
  d. Browse to the project’s API page https://console.cloud.google.com/apis/dashboard and click on “Enabled APIs & services” in the sidebar, then click enable all maps API.
  e. Browse to the project’s credentials page https://console.cloud.google.com/apis/credentials and click “create credentials” to create a new API key.

***Note:*** this step is optional and needed only when making additions to the network in the model. Our code used for the geocoding work uses the Google Maps API. Use of other services will require substantial rewriting of this part of the code.

## Key resources table

**Table:** 

REAGENT or RESOURCE	SOURCE	IDENTIFIER
**Deposited data**		

The installation-level China coal model IL-CCM	https://zenodo.org/record/7222764	https://doi.org/10.5281/zenodo.7222764

**Software and algorithms**		

R version 4.0.5	https://cran.r-project.org/	v4.0.5
RStudio Desktop	https://www.rstudio.com/products/rstudio/	V1.2.5033
R build tools version 4.0	https://cran.r-project.org/bin/windows/Rtools/	v4.0
here	https://cran.r-project.org/web/packages/here/index.html	v1.0.1
readr 2.0.2	https://cran.r-project.org/src/contrib/Archive/readr/	v2.02
readxl 1.3.1	https://cran.r-project.org/src/contrib/Archive/readxl/	v1.3.1
tidyverse 1.3.0	https://cran.r-project.org/src/contrib/Archive/tidyverse/	v1.3.0
stringr 1.4.0	https://cran.r-project.org/src/contrib/Archive/stringr/	v1.4.0
formattable 0.2.1	https://cran.r-project.org/src/contrib/Archive/formattable/	v0.2.1
ggplot2 3.3.6	https://cran.r-project.org/src/contrib/Archive/ggplot/	v3.3.6
ggpubr 0.4.0	https://cran.r-project.org/src/contrib/Archive/ggpubr/	v0.4.0
RColorBrewer 1.1.2	https://cran.r-project.org/src/contrib/Archive/RColorBrewer/	v1.1.2
scales 1.1.1	https://cran.r-project.org/src/contrib/Archive/scales/	v1.1.1
geosphere 1.5.14	https://cran.r-project.org/src/contrib/Archive/geosphere/	v1.5.14
ggmap 3.0.0	https://cran.r-project.org/src/contrib/Archive/ggmap/	v3.0.0
ggspatial 1.1.6	https://cran.r-project.org/src/contrib/Archive/ggspatial/	v1.1.6
sf 1.0.7	https://cran.r-project.org/src/contrib/Archive/sf/	v1.0.7
rgeos 0.5.9	https://cran.r-project.org/src/contrib/Archive/rgeos/	v0.5.9
maps 3.4.0	https://cran.r-project.org/src/contrib/Archive/maps/	v3.4.0
rnaturalearth 0.1.0	https://cran.r-project.org/src/contrib/Archive/rnaturalearth/	v0.1.0
rnaturalearthdata 0.1.0	https://cran.r-project.org/src/contrib/Archive/ rnaturalearthdata/	v0.1.0
Anaconda 3 v2022.05	https://www.anaconda.com/products/distribution	v2022.05
Python 3.6.10	https://anaconda.org/conda-forge/python/files?version=3.6.10	v3.6.10
pandas 1.1.1	https://anaconda.org/conda-forge/pandas	v1.1.1
numpy 1.19.1	https://anaconda.org/cctbx202008/numpy	v1.19.1
xlrd 1.2.0	https://anaconda.org/conda-forge/xlrd	v1.2.0
spyder 4.1.5	https://anaconda.org/conda-forge/spyder	v4.1.5
PuLP 2.3	https://anaconda.org/conda-forge/pulp	v2.3
CPLEX Solver	https://www.ibm.com/products/ilog-cplex-optimization-studio	v20.1.0

**Other**		

AWS Sagemaker	https://aws.amazon.com/sagemaker/	N/A
Microsoft Azure ML	https://azure.microsoft.com/en-au/services/machine-learning/	N/A
Google Maps api	https://developers.google.com/maps	N/A

## Step-by-step method details

### Insert coal mine data

**Timing: 1 h**

The IL-CCM code and data repository is complete apart from the coal-mine level data, which is proprietary and has been censored. This step is to replace the censored data with real data.

The original model was created with the coal supply dataset from Wood Mackenzie.^19^ Inserting this same data is straightforward as the layout and variable naming from this dataset have been preserved.

The user can also use their own coal mine data, or from any other commercial supplier, but inserting such data into the model will require additional steps, as described below.

1. Option 1: when using the coal supply dataset from Wood Mackenzie.^19^
  a. Browse to the Wood Mackenzie data dashboard, and download the full set of production cost data, production data, and coal quality data by mine and year in csv format, and download the global supply curve data.
    ***Note:*** We have provided spreadsheet files that allow the user to copy paste the entirety of the original Wood Mackenzie data into a sheet called ‘input’. The spreadsheets have formulas that automatically find the correct values on those ‘input’ sheets and transform these into a data layout as expected by the model.
  b. For the files in “∼/China-coal-model-star-protocol/1 input/WoodMac data - template”:
    i. Copy the entirety of production cost data into the sheet ‘input’ in the file “selected china coal mine cost data woodmac -- CENSORED.xlsx”.
    ii. Copy the entirety of production data into the sheet ‘input’ in the file “selected china coal mine prod data woodmac -- CENSORED.xlsx”.
    iii. Copy the entirety of coal quality data into the sheet ‘input’ in the file “selected coal quality data by mine and type -- CENSORED.xlsx”.
    iv. Copy the entirety of the global cost curve data into the sheet ‘input’ in each of the following 3 files “Seaborne Export Thermal - non energy adjusted Worksheet -- CENSORED.xlsx”, “Seaborne Export Met Worksheet -- CENSORED.xlsx”, “Australia met coal worksheet -- CENSORED.xlsx”.
    v. Copy all of the files in the folder “∼/China-coal-model-star-protocol/1 input/WoodMac data - template” into the folder “∼/China-coal-model-star-protocol/1 input/WoodMac data”, overwriting the files there in the process.
2. Option 2: when using mine-level data from other sources.
  ***Note:*** We have provided spreadsheet files with templates including a small set of example entries for each sheet that needs to be adjusted. Each of these template files contains a sheet named ‘possible values’ to explain restrictions on the values in each column. These spreadsheets are included in “∼/China-coal-model-star-protocol/1 input/alternative coal data - template”.
  a. Insert coal mine data as follows:
    i. In the file “selected coal quality data by mine and type -- CENSORED.xlsx”: for every mine, define its name. Define whether its product type is thermal or metallurgical coal.
      ***Note:*** For metallurgical, also define product sub-type (hard coking coal, semi-soft coking coal, or pulverized coal for injection. For thermal coal, a product subtype can be included but this information is not used. For thermal coals, define a calorific value either as net as received (SE nar) or as gross as received (SE gar), or both. For metallurgical coals define their coking strength after reduction (CSR) number.
    ii. In the file “selected china coal mine prod data woodmac -- CENSORED.xlsx”: for every mine, define its name. Define whether its product type is thermal or metallurgical coal.
      ***Note:*** For metallurgical, also define product sub-type (hard coking coal, semi-soft coking coal, or pulverized coal for injection. For thermal coal, a product subtype can be included but this information is not used. For each year considered in your analysis, define an operating status and a production capacity for each mine.
    iii. In the file “selected china coal mine cost data woodmac -- CENSORED.xlsx”: for every mine, define its name. Define whether its product type is thermal or metallurgical coal.
      ***Note:*** For metallurgical, also define product sub-type (hard coking coal, semi-soft coking coal, or pulverized coal for injection. For thermal coal, a product subtype can be included but this information is not used. For each year considered in your analysis, define the total production cost, and where this data is available, royalties, product transport cost, and port fees.
    iv. In the file “Seaborne Export Thermal - non energy adjusted Worksheet -- CENSORED.xlsx”, in the sheet “worksheet navo mines connectors”: for every thermal coal mine supplying the seaborne market, define its name, and the country and 3 letter iso country code it is located in. Also define its production capacity, production cost, and the calorific value of its output.
      **CRITICAL:** The destination node name (column dest-node_name) has to list the node that connects these foreign mines to the rest of the node-and-link network. There is one such node for each country, with values provided in the ‘possible values’ sheet. Do the same for all Russian thermal coal mine connected to the Chinese market via rail or road connections, in the sheet “worksheet ovld mines connectors”.
    v. In the file “Seaborne Export Met Worksheet -- CENSORED.xlsx”, do the same as in last step, but for metallurgical coal mines. Define their coal subtype (hard coking, semi-soft coking, or PCI), and not their calorific value. Exclude Australian mines in this sheet.
    vi. In the file “Australia met coal worksheet -- CENSORED.xlsx”, do the same as in last step, but for Australian metallurgical coal mines.
    vii. Copy all files in the folder “∼/China-coal-model-star-protocol/1 input/alternative coal data - template” into the folder “∼/China-coal-model-star-protocol/1 input/WoodMac data”, overwriting the files there in the process.
  b. Connect the mines defined in the above step to the rest of the node-and-link network.
    ***Note:*** For this step, we have provided template files with a small set of example entries. Each of these template files contains a sheet named ‘possible values’ to explain restrictions on the values in each column. Adjust as follows:
    i. From each of the files created in step a above, collect all values in the column ‘orig_node_name’, and create a list with unique values of node names (i.e., each node appearing only once) in the file “∼/China-coal-model-star-protocol/1 input/helper files/mine node list helper.xlsx”.
      ***Note:*** the above mentioned file in the code and data repo contains exactly such a list for the node names as used in all of the files described in step a above.
    ii. Copy the list of mine node names from step i into the file “∼/China-coal-model-star-protocol/1 input/alternative coal data - template/helper files/node location helper.xlsx”, sheet “loclist”, pasting the list in column A, row 11438 onwards (highlighted in green in the spreadsheet).
    iii. For each mine node in step ii, add latitude and longitude.
    iv. Copy the list of mine node names from step i into the file “∼/China-coal-model-star-protocol/1 input/alternative coal data - template/helper files/mine name to location.xlsx”, sheet “Sheet1”, pasting the list in column B, row 2 onwards (highlighted in green in the spreadsheet).
    v. For each mine node in step iv, add the location, which is either the country name, or for mines in China, the name of the province. For mines in China and Mongolia, also select the corresponding basin name.
      ***Note:*** A list of self-explanatory basin names is provided in the sheet “possible values”.
    vi. For those mine nodes located in China, copy the list of node names and basins from step iii into the file “∼/China-coal-model-star-protocol/1 input/alternative coal data - template/helper files/basin and mine names.xlsx”, sheet “china mines”, pasting the node name list into column B, row 2 onwards and the corresponding basin names into column C, row 2 onwards (highlighted in green in the spreadsheet). The rest of sheet automatically updates.
    vii. For those mine nodes located in Mongolia, copy the list of node names from step i into the file “∼/China-coal-model-star-protocol/1 input/alternative coal data - template/network connector files/ international mongolia russia interconnectors cities.xlsx”, sheet “international”, pasting the node name list into column B, row 44 onwards (highlighted in green in the spreadsheet).
    viii. For each mine node in step vii, indicate to which node it should be connected (column dest_node_name), picking from the list of nodes provided in the sheet “possible values”.
      ***Note:*** These destination nodes are all cities or railway stops along the border with Mongolia. More precise information on each is provided in the sheet “dest node description”.
    ix. For each mine node in step vii, Also define whether the connection type between the mine and destination nodes is via rail or road, and what distance the link represents.
    x. Copy the contents of the folder “∼/China-coal-model-star-protocol/1 input/alternative coal data - template” into the folder “∼/China-coal-model-star-protocol/1 input/”, overwriting the files there in the process.

### Adjust scenario inputs and create model build files

**Timing: 1–2 h**

These are instructions on how the user may define their own scenario settings for coal production, consumption and transport infrastructure capacity. Running the build preparation script (step 9 below) gathers the scenario settings from their respective spreadsheet files and processes these into model build files which are in the correct format to be read by the optimization script used in the next step.

3. Open the file “∼/China-coal-model-star-protocol/1 input/input file details.xlsx”.

***Note:*** This file contains explanations on the calculations made in each of the spreadsheets in the folder “∼/China-coal-model-star-protocol/1 input”, including on the values to be adjusted for scenario settings.

4. Adjust demand for coal through 2030 for different industries using the files in “∼/China-coal-model-star-protocol/1 input/energy demand files”.

***Note:*** Demand levels can either be set at national levels, with an automatic distribution to provincial-level demand calculated by these sheets, or the user can adjust provincial-level demand directly.

5. Adjust production capacity of coal-fired power plants and steel plants through 2030 using the files in “∼/China-coal-model-star-protocol/1 input/Global Energy Monitor data”.
6. Adjust transport capacity of the railway network through 2030 using the file “∼/China-coal-model-star-protocol/1 input/rail network/rail line capacities.xlsx”.
7. Adjust annual throughput capacity of coal ports through 2030 using the file “∼/China-coal-model-star-protocol/1 input/port data/port loc and capa consolidated sources.xlsx”.
8. Adjust transmission capacity of the UHV network through 2030 using the file “∼/China-coal-model-star-protocol/1 input/network connector files/UHV overview.xlsx”.
9. Run the script “∼/China-coal-model-star-protocol/1 Build preparation script.R”; this will take adjusted input files in “∼/China-coal-model-star-protocol/1 input” and summarize these into model build files, which are saved into “∼/China-coal-model-star-protocol/2 build”.

### Run the script that creates the optimization problem file

**Timing: 10 h**

This step will take your scenario build files, and process it these with the Linear Programming modeler Package “PuLP” (https://pypi.org/project/PuLP/). You can do this on a desktop machine, but it is recommended to be done using an online cloud computing service. These steps will create a problem file (extension “.lp”) to be used in a solver in the next step.

10. Option 1: Create the problemfile with MS Azure.
  a. Browse to https://portal.azure.com/#home and click on the Azure Machine Learning instance created under “sign up for and set up a cloud computing account”.
  b. Click “launch studio”.
  c. Click “start now” under “Notebooks”.
  d. In the file folder structure, click on whatever folder you want to run the code in.
  e. In that folder, create two subfolders called “builds” and “problemfiles”.
  f. In that same folder, upload the file “∼ /China-coal-model-star-protocol/2c Create problem file on MS Azure ML.ipynb".
  g. In the folder “builds”, upload all the .csv files from the folder “∼ /China-coal-model-star-protocol/2 build".
  h. Open the file “2c Create problem file on MS Azure ML.ipynb” on Azure, select the year you want to run the model for and fill that in line 32, i.e.:
    >selected_year = 2025
  i. Click on “Compute” under “Manage” in the sidebar and click “new”. Choose your options.
    ***Note:*** We’d recommend a “compute optimized” instance with at least 16 virtual CPU and 32 GB of memory, i.e., an instance of type “Standard_F16s_v2” or better.
  j. Click on “Notebooks” under “Author” in the sidebar to go back to the optimization model script.
  k. Click on “+ Code” under the code cell and type:
    > pip install pulp
  l. Click “Run cell” next to the cell created in step k.
  m. Click “Run cell” next to the first cell.
  n. The problemfile created by these steps will appear in the folder “problemfiles” after about 10 h; download it and copy it to “∼ /China-coal-model-star-protocol/4 solutionfiles".
11. Option 2: Create the problemfile with AWS Sagemaker.
  a. Browse to https://aws.amazon.com/sagemaker/ and login.
  b. Click “Services”, and then “S3”.
  c. Click “create bucket”, use a bucket name “sagemaker-studio-SOMETHING”, i.e., anything starting with “sagemaker-studio”; this allows easier access to the bucket from sagemaker studio.
  d. In this bucket, create two subfolders called “builds” and “problemfiles”.
  e. In the folder “builds”, upload all the .csv files from the folder “∼ /China-coal-model-star-protocol/2 build".
  f. Click “Services”, “Amazon Sagemaker”, then “Sagemaker dashboard” in the sidebar, then “Open Sagemaker Domain”, then “launch app”, then “Studio” from the dropdown.
  g. In the Sagemaker studio, click “upload files”, and upload the file “∼ /China-coal-model-star-protocol/2b Create problem file on AWS Sagemaker.ipynb".
  h. Double click the uploaded file, choose image “Data Science”, Kernel “Python 3”, Start-up script “No script” when promoted, and click select.
  i. Click “Select instance” at the top and select your preferred type of instance.
    **CRITICAL:** You must have previously requested access to this type of instance. How to get that access is explained in step 8 under “sign up for and set up a cloud computing account”.
  j. Click on the + icon to insert a cell below, and type:
    > pip install pulp
  k. Click on this cell and click “Run cell” from the menu at the top.
  l. Click on the first cell and fill in the bucketname from step c where it reads:
    > bucket ='sagemaker-studio-SOMETHING'
  m. Select the year you want to run the model for and fill that in where it reads:
    > selected_year = 2025
  n. Click “Run cell” in the menu at the top.
  o. The problemfile created by these steps will appear in the folder “problemfiles” after about 10 h; download it and copy it to “∼ /China-coal-model-star-protocol/4 solutionfiles".
12. Option 3: (Not recommended): Create the problemfile on your desktop machine.
  a. Launch an anaconda prompt and type.
    > conda activate china-coal-imports
  b. Launch spyder by typing.
    > spyder
  c. Click open file and browse to “∼ /China-coal-model-star-protocol/2a Create problem file locally.py".
  d. Fill in the correct file location of the code and data repository in line 15, e.g.:
    > currentDirectory = "∼/China-coal-model-star-protocol/"
  e. Select the year you want to run the model for and fill that in line 12, e.g.:
    > selected_year = 2025
  f. Press F5 to run the file.
  g. The problemfile created by these steps will appear in the folder “∼/China-coal-model-star-protocol/3 problemfiles" after a given period of time, depending on the computing power of your desktop machine, between circa 10 h to many days.

### Solve the optimization problemfile

**Timing: 10 m**

This step will take your problem file, which is a text file describing the optimization problem, and find the minimal cost solution to the problem. This solution file will be saved to “∼ /China-coal-model-star-protocol/4 solutionfiles”.

***Note:*** There are several possible solvers to use. Below are instructions for two popular alternatives. The CPLEX solver included with the CPLEX Optimization Studio is typically several times faster than the Coin-or branch and cut (CBC) solver, and can be 10–100 faster when compared with other open source solvers. IBM offers a free license for the CPLEX Optimization Studio, but only for academic users, however. The CBC solver is an open source and completely free alternative.

13. Option 1: with CPLEX Studio.
  a. Open CPLEX Optimization Studio.
  b. Type below code, adjusting for the file name for the problem file you created in the previous step:
    > read "∼\China-coal-model-star-protocol\3 problemfiles\prob v13 baseline 2019.lp"
    > opt
    > set logfile "∼\China-coal-model-star-protocol\4 solutionfiles\solution v13 baseline 2019.txt"
    > display solution variables -
14. Option 2: with python and the Coin-or branch and cut solver (https://github.com/coin-or) included in PuLP.
  a. Launch an anaconda prompt and type.
    > conda activate china-coal-imports
  b. Launch spyder by typing.
    > spyder
  c. Click open file and browse to “∼/China-coal-model-star-protocol/3a Solve with python.py".
  d. Adjust the problem file and solution file name and location in lines 10 and 12.
  e. Run the script.

### Process the solution file

**Timing: 1 m**

This step will take your solution file, and translate it into a set of easily readable spreadsheets, which are saved to “∼/China-coal-model-star-protocol/5 solution processed”.

15. Open and run the file “∼/China-coal-model-star-protocol/4 Process solution flows cons.R”.
16. Open and run the file “∼/China-coal-model-star-protocol/4 Process solution national.R”.

### Prepare visualizations of updated scenario runs

**Timing: 10 m**

This step will take the spreadsheets with results from last step, and create visualizations of your scenario settings.

17. Open and run each of the six R scripts in the folder “∼/China-coal-model-star-protocol/ 6 figure prep scripts”.

### Optional: Expanding or adjusting the network in the model

**Timing: 1–10 h**

This step is only needed when the user wants to expand the node-and-link model. If the user simply wants to adjust transport capacities of links currently included in the model, this can be done by following the instructions in step 6. If the user wants add nodes or links to the network, it is important that these are included into the full node-and-link model in the correct way. We cannot provide precise protocol steps for every possible expansion of the model, but provide a number of generic steps and scripts to do this below.

As an example, we have included the exact data and code that we used to identify driving distances between each city and its nearest neighboring cities, in the folder “∼/China-coal-model-star-protocol/9 geocoding network additions”.

18. Geocode your nodes.
  ***Note:*** This step finds the latitude and longitude of a description of a location. This can be a street address, the name of a power plant or steel plant, or in our example here, the names of cities. Include as much detail on the location to geocode as you can. If you already have latitude and longitude data for your locations, you can skip this step.
  a. Input your locations to geocode into the file “∼/China-coal-model-star-protocol/9 geocoding network additions/locations to geocode.xlsx”, column A.
  b. Open script “∼/China-coal-model-star-protocol/9 geocoding network additions/geocode locations.R” and copy your Google Maps api credentials into line 19:
    >register_google(key = "your_key", write = TRUE)
  c. Run this script; this saves location names with latitude and longitude data into the file “∼/China-coal-model-star-protocol/9 geocoding network additions/locations geocoded.xlsx”.
19. Determine geodesic distances.
  ***Note:*** This step takes the geocoded locations from step 18, and creates a matrix with a combination of each location with all other locations in list of nodes from step 18. This is used to calculate distances from each node to every other node, using the latitude and longitude data of each point determined in step 18, as geodesic or “as the crow flies” distances. For each node, it retains links with the 12 nearest other nodes. For nodes even further away, we consider that these can be reached via other nodes. This ensures that each node is connected, whilst reducing the total number of links.
  a. Copy the data (location names with latitude and longitude) in the file “∼/China-coal-model-star-protocol/9 geocoding network additions/locations geocoded.xlsx”, created in step 18, into the files for origins “∼/China-coal-model-star-protocol/9 geocoding network additions/origins.xlsx” and the file for destinations “∼/China-coal-model-star-protocol/9 geocoding network additions/destinations.xlsx”.
    ***Note:*** The example files currently included with the data and code repository contain a list of Chinese cities and their latitude and longitude.
  b. Run the script “∼/China-coal-model-star-protocol/9 geocoding network additions/calculate geodesic distances.R”; this calculates “as the crow flies” distances between all node pairs and saves this info in “∼/China-coal-model-star-protocol/9 geocoding network additions/geodesic distances all.csv”.
20. Find driving distances.
  ***Note:*** This step takes the pairs of nodes that are relatively close to each other, as determined with “as the crow flies” distances in step 19, and finds the real world driving distance between these node pairs.
  a. Open script “∼/China-coal-model-star-protocol/9 geocoding network additions/geocode distances.R” and copy your Google Maps api credentials into line 21:
    >register_google(key = "your_key", write = TRUE)
  b. Run this script; this saves location names with geodesic as well as driving distances into the file “∼/distances 12 nearest geodesic and geocoded.csv”.
21. Insert these new connections in the appropriate file in the folder “∼/China-coal-model-star-protocol/1 input/network connector files”.

## Expected outcomes

The expected outcome with each run is 1) a problem file; 2) a solution file; 3) a set of spreadsheets with coal suppliers and coal flows; and 4) a set of visualizations of these model outcomes. The IL-CMM was built specifically to determine levels and origin of coal imports, at given levels of Chinese demand for coal, and with constraints on mine-level production capacity and transport capacity of different types of transport infrastructure. Model outputs are therefore focused on visualizing the origin of supply, the networks used to get to the final source of consumption, and changes in flows of coal under different scenario settings.

The code and data repository includes 9 problem files; for each of the years 2015–2019, and a low and high demand scenario for 2025 and 2030. These demand scenarios correspond with the “stated policies” and “sustainable development” scenarios from the IEA’s World Energy Outlook.^20^

Figure 2 includes the example visualization of model results for the years 2015–2019, and a low and high demand scenario for 2025 and 2030. For thermal coal, these results shows that imports have been fairly stable over the period 2015–2019, but are expected to come down sharply over the period to 2025, even in the scenario of continued high Chinese coal demand. Indonesian imports of thermal coal are expected to fall most sharply, both in total volume as well as in percentage terms, followed by Australian imports. This is due mostly to improvements in transport infrastructure, in particular rail lines that have become operational over 2020–2022, and some that are in currently in advanced stages of construction. These new rail projects strongly lower the cost of delivery of coal from domestic mines. The reduced demand for imported coal is further due a continuing improvement in power plant efficiency. For more details, see the original analysis in Gosens et al.^1^ For coking coal, there has been a steady rise of imports that have tracked closely with total volumes of Chinese steel output. Over the period through to 2025 and beyond, total Chinese coking coal consumption is expected to fall, as crude steel production is expected to have peaked. A simultaneous increase in the availability of steel scrap improves the supply of secondary steel (made from recycled scrap) and reduces the need for primary steel (made with iron ore and coking coal). Total imports of coking coal are still expected to rise, as a new rail line connects the steel making heartland of China directly with a new coking coal mine in Mongolia, which produces high quality and low cost coking coal. The additional imports form Mongolia are expected to push out imports from Australia (Figure 2).

**Figure 2:**
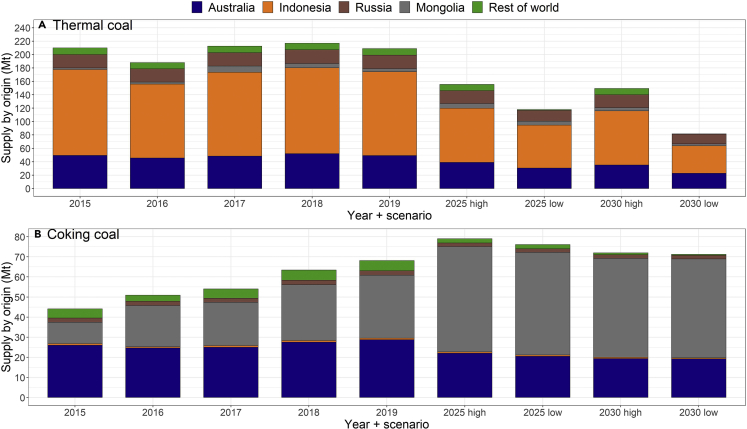
Origin of Chinese coal imports for 2015–2019, with high and low demand scenario forecasts for 2025 and 2030 Thermal (A) and coking (B) imports are shown.

Figure 3 gives an example visualization of the flows of coal via the different transport networks in the IL-CCM. Results displayed are model results for the cost-optimal flow of coal from mines to final point of consumption in the year 2019. They show the large flows of coal from the mining regions of in the north of China (primarily Shaanxi, Shanxi, and Inner Mongolia) flowing east via rail (plot a) towards ports in the Bohai Bay, where they are loaded for transport via ship to coastal provinces in the South (plot c). The same plot shows that seaborne imports of coal predominantly flow to consumers in the Southern coastal provinces. Those model results closely resemble actual flows in coal in China and therefore give additional confidence in the accuracy of the IL-CCM model results.^1^

**Figure 3:**
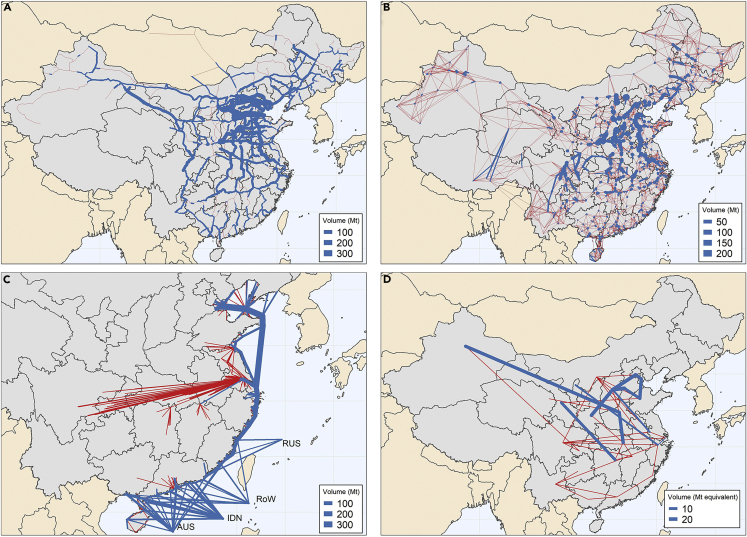
Flows of coal via the different transport networks in the IL-CCM; results for 2019 Results are shown for transport via rail (A), via truck (B), via ship (C), or as electricity transmitted via UHV lines (D). Red lines are links in the transport networks that are not used in the least-cost solution as determined by the optimization model.

Figure 4 provides changes in coal consumption in 2025 versus 2019, for different coal types and for different demand scenarios, at the level of individual power or steel plants. This plot shows that changes in plant-level consumption of coal between 2019 and 2025, with either reductions or increases, for both thermal and coking coal, are distributed roughly equally across the different provinces of China. As can be expected, plant-level reductions in consumption dominate in both the high consumption “stated policies scenario” and low consumption “sustainable development scenario”, with stronger reductions in the latter (Figure 4).

**Figure 4:**
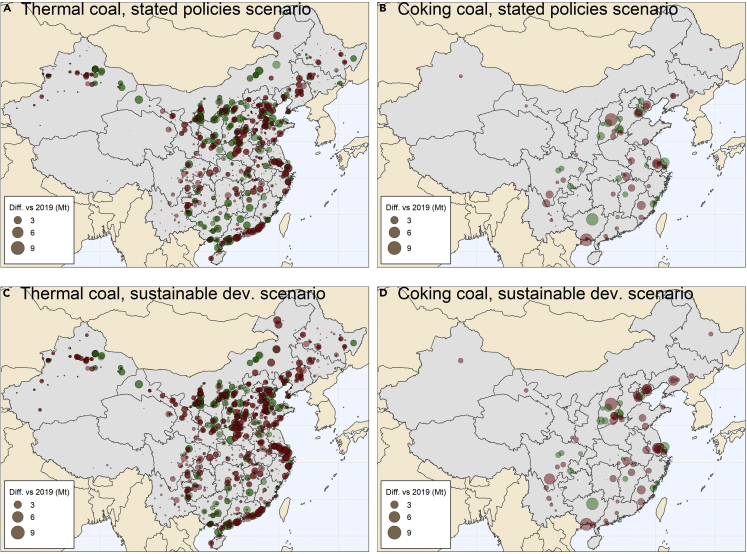
Plant-level changes in coal consumption in 2025 versus 2019, for different coal types and for different demand scenarios Green circles indicate an increase in coal consumption between 2019 and 2025; red circles indicate a decrease in coal consumption between 2019 and 2025.

Figure 5 provides an example visualization of changes in flows of coal over the transport networks included in the IL-CCM in 2025 versus 2019. For thermal coal, the clearest result is the addition of the HaoJi rail line, which shows up as a dark green streak in plots a and c in Figure 5. This rail line went into operation in September of 2020 and is capable of transporting 200 Mt of coal from the key coal mining region around Ordos in Inner Mongolia, to China’s central provinces (so flowing north to south). This means less coal needs to flow west towards Bohai Bay and then south via ship, drastically reducing the cost of coal deliveries, as was the intention of the project.^7^ The flows of seaborne imports of coal appear very light red, as these changes are small compared to changes in coal from domestic sources, but still represent a significant change to the total volume of imported thermal coal (compare Figure 2). For coking coal, there is a clear reduction in flows of coal throughout most of China in both the scenarios with high (plot b) and low (plot d) demand. This illustrates that reduced demand for primary steel leads to lower consumption and therefore shipments of coking coal. Reductions in shipments from both domestic and seaborne suppliers are further exacerbated by increases in coking coal shipments from neighboring Mongolia, showing up as the only green streak in both plots b and d in Figure 5.

**Figure 5:**
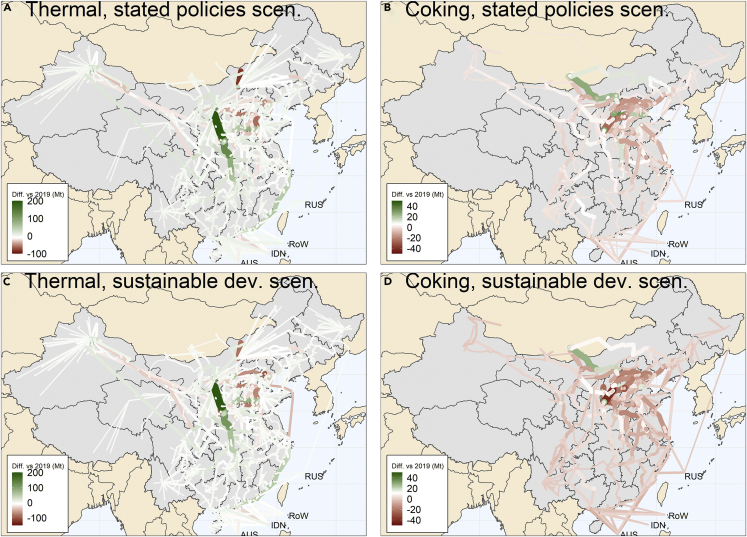
Changes in flows of coal transported via the detailed network of the IL-CCM, in 2025 versus 2019, for different coal types and for different demand scenarios Green lines indicate an increase in coal transported over a link between 2019 and 2025; red lines indicate a decrease in coal transported over a link between 2019 and 2025.

## Limitations

The current model is a highly detailed model of China’s coal market. The demand and supply conditions in the rest of the world are included in very simplified form only, and cannot be adjusted. The model is also purely focused on coal consumption; it does not include cost-optimization between different sources of electricity and instead uses exogenous input from user defined demand scenarios to determine cost-optimal supply of that demand.

## Troubleshooting

### Problem 1

A package throws a certain error in R or Python.

### Potential solution

It is recommended to use the environment files for both R and python provided with the code and data repository. These have been tested to run as a self-contained package. Instructions on how to use either of these environment files are provided under step “install the required software”.

### Problem 2

The solver reports that the problem is infeasible.

### Potential solution

This is a generic issue that could happen when defining problem files. It indicates an incompatibility between different constraints in the model. Investigate by opening the problem text file and finding the definition of the constraint that the CPLEX Optimization Studio reports as causing the infeasibility. A number of likely causes are:

• You’ve set demand levels for a certain type of coal at level exceeding the production capacity of all the mines in the model. This is an issue especially with PCI, which has the least supply slack in the model.
• You’ve set provincial level demand for coal-fired power or steel at levels exceeding the provincial-level production capacity of coal-fired power plants or steel plants.
• You’ve set a reduction in transport network capacities that disconnects different parts of the network from each other. This is unlikely as almost all nodes can also be reached by trucking networks.
• A special case is that the provincial-level city of Beijing has no operational power plants in the dataset after 2029; any coal-fired power demand must be delivered via UHV networks. Removing the UHV network whilst simultaneously setting a non-zero coal-fired power demand in Beijing will cause infeasibility.

The solution is to investigate whether and which demand, supply, and transport constraint cause the infeasibility and adjust scenario settings.

### Problem 3

When running the script in AWS Sagemaker it reports an error loading the files from the S3 bucket. This is likely due to a version of boto3 (the software package that allows python to communicate with other components of the AWS infrastructure such as the file storage system), as installed on AWS Sagemaker by default, that is incompatible with the provided script.

### Potential solution

Uninstall the incompatible version of boto3 and its dependencies, and install a version that does work. To do so, run below code in a new cell in your jupyter notebook:

> pip uninstall -y boto3

> pip uninstall -y botocore

> pip uninstall -y aiobotocore

> pip install boto3==1.17.106 s3fs pulp mipread

## Resource availability

### Lead contact

Further information and requests for resources should be directed to and will be fulfilled by the lead contact, Jorrit Gosens (jorrit.gosens@anu.edu.au).

### Materials availability

This study did not generate new unique reagents.

## Data Availability

All data and code for this protocol have been made available via zenodo: https://doi.org/10.5281/zenodo.7222764.

## Supplemental information

Methods S1. Model description, related to before you begin
