# Supplementary material for: A protocol to determine the least cost supply of coal to China with an installation-level optimization model
Source: STAR Protoc. 2022 Nov 23;3(4):101873. doi: 10.1016/j.xpro.2022.101873 (PMC9700009; doi:10.1016/j.xpro.2022.101873)
Supplement: Methods S1. Model description, related to before you begin [file mmc1.pdf]

Methods S1: Model description, related to Before you begin

The Installation-Level China Coal Model (IL-CCM) is a linear optimization model that determines the cost-optimal supply of thermal and coking coal to Chinese power plants and steel plants. It seeks to minimize the total cost of production and transport of coal in order to satisfy an exogenously determined level of coal-fired power generation and steel demand.

The core component of the model is a node-and-link model that represents China's coal production, transport, and consumption system. It includes location and technical detail for each coal mine or mine cluster, both those in China and foreign mines supplying the global seaborne coal market, with detail on production capacity, type and quality of coal produced, and production cost. This data is sourced from Wood Mackenzie.[19] The model also includes information on all operational and planned coal-fired power and steel plants in China, with details on location, access to rail or port facilities, production capacity, and power generation efficiency. This data is derived from the Global Energy Monitor.[21] For rail networks, we rely on an open source called the China Railway Map (http://cnrail.geogv.org/), which in turn builds on the OpenStreetMap (openstreetmap.org) for location of rail lines and railway stops, and Wood Mackenzie data for transport capacity info.[19] We use data from Kpler (https://www.kpler.com/) to identify location and annual handling capacity of individual ports, including for port facilities at individual power and steel plants. We use a public list of Chinese cities and the Google Maps api (https://developers.google.com/maps) to determine driving distances between cities, and between cities and mine, railway, port, and power or steel plant nodes. We get information on location and transmission capacity of China's Ultra-High Voltage transmission network from a report by the Lantau Group.[22] For more details on the data collection exercise to construct this network, see the analysis where this model was first used in Gosens et al.[1] Table 1 provides an overview of the types of nodes and links in the model.

Table 1. Components of the node-and-link network in the IL-CCM

Nodes of each type:	Number
Power plants/units	1,680/3,364
Steel plants	276
Railway stops	4,743
City centers	685
Navigation waypoints	103
Ports	483
Coal mines in China/abroad	174/542
Prov. Level power/steel demand centers	30/28
Total nodes	12,161
Links of each type:	Number
Railway	9,938
Inter-city road network	6,132
Other road connections	22,790
River or ocean connections	1,432
UHV network	62
Mine-mouth connections	43
Total links	40,396
Total of all nodes and links	52,557

The optimization model is formulated as follows in the set of equations below. Subscripts in these equations refer to model sets. Model sets, parameters, and variables are defined in Tables 4-6. For more details on the logic of the optimization model, see the analysis where this model was first used in Gosens et al.[1]

The objective function minimizes the total production and transport of coal, plus the cost of transmission of electricity via the inter-provincial UHV network. Transport costs include fixed handling costs for when a shipment of coal is transferred between different modes of transport, plus a variable distance-based cost component. Transmission of electricity over the UHV network is represented as transport of coal over electricity lines, with energy content converted to electrical energy and considering a fixed percentage of (pre-calculated) distance-based line losses. Formally:

$$Minimize \sum_{i,c,t} supply\_Mt_{i,c,t} \times prod\_cost_{i,c,t} \times 1e6 +$$
$$\sum_{i,c,t} transp\_Mt_{i,j,m,t} \times \left(handling\_cost_{k,k\prime,t} + transp\_cost_{m,t} + distance_{i,j}\right) \times 1e6 +$$
$$\sum_{i,c,t} transp\_Mt_{i,j,v,t} \times CV_v \times kcal\_to\_PJ \times conv\_eff_{i,j} \times transm\_cost_{i,j,t} \times 1e9$$

The optimization in this objective is subject to a set of constraints. First, mine nodes cannot supply coal of any type above their production capacity for that type of coal, and supply cannot be negative:

$$0 \geq supply\_Mt_{i,c,t} \leq prod\_capa_{i,c,t}$$

A mass balance constraint states that total volumes of coal flowing out of a node may be no greater than the total volume of coal supplied by a node plus coal flowing into that node, of each type of coal:

$$supply\_Mt_{i,c,t} + \sum_{j,i,c,t} transp\_Mt_{j,i,c,t} \geq \sum_{i,j,c,t} transp\_Mt_{i,j,c,t}$$

An energy balance constraint states that the total energy content demanded in a node plus energy content of all types of coal flowing out of that node may be no greater than then the energy content of the coal flowing into that node and supplied by that node:

$$supply\_Mt_{i,c,v,t} \times CV_v \times 1e9 \times kcal\_to\_PJ +$$
$$\sum_{j,i,c,v,t} transp\_Mt_{j,i,c,v,t} \times CV_v \times 1e9 \times kcal\_to\_PJ \geq$$
$$elec\_demand_{i,t} + other\_demand_{i,t} + \sum_{i,j,c,v,t} transp\_Mt_{i,j,c,v,t} \times CV_v \times 1e9 \times kcal\_to\_PJ$$

A transport constraint states that the total amount of coal of all types transported over any transport link (railways etc.) cannot exceed that link's annual transport capacity:

$$\sum_{i,j,c,t} transp\_Mt_{i,j,c,t} \leq transp\_capa_{i,j,t}$$

A port capacity constraint states that the total amount of coal handled in each port, i.e., either loaded or unloaded, cannot exceed that port's annual handling capacity:

$$\sum_{p,j,c,t} transp\_Mt_{p,j,c,t} \leq port\_capa_{p,t}$$

An electric power generation capacity constraint states that the energy content of all types of coal flowing into power plants nodes, multiplied by the power plant node's conversion efficiency, cannot exceed the power plant's generation capacity:

$$\sum_{i,j,c,v,t} transp\_Mt_{i,j,c,v,t} \times CV_v \times 1e9 \times kcal\_to\_PJ \times conv\_eff_{i,j} \leq elec\_capa_{i,j,t}$$

A steel plant capacity constraint states that the total amount of coking coal transported to a steel plant, multiplied by 0.966, cannot exceed the steel plant's steel production capacity. This represents that we use a conversion factor of 966 kg of coking coal needed to produce one ton of steel:

$$\sum_{p,j,c,t} transp\_Mt_{p,j,c,t} \leq stpt\_capa_{s,t} \times 0.966$$

A trio of coking coal mix constraints further specifies that a steel plant has to use a specific mix of coking coal to produce one ton of steel. The 966 kg of coking coal in above equation has to be made up of 581 kg of Hard Coking Coal (HCC), 176 kg of Semi-soft Coking Coal (SCC) and 179 kg of Pulverized Coal for Injection (PCI). This is represented by stating that amount of coal flowing into a node, multiplied by a dummy variable indicating whether the coal is of a HCC, SCC, or PCI types, respectively, has to be at least equal to steel demand multiplied by 0.581, 0.176, or 0.179, respectively:

$$\sum_{s,j,c,t} transp\_Mt_{s,j,c,t} \times HCC_c \geq steel\_demand_{j,t} \times 0.581$$

$$\sum_{s,j,c,t} transp\_Mt_{s,j,c,t} \times SCC_c \geq steel\_demand_{j,t} \times 0.176$$

$$\sum_{s,j,c,t} transp\_Mt_{s,j,c,t} \times PCI_c \geq steel\_demand_{j,t} \times 0.179$$

Table 2. Model variables in the IL-CCM

Variable	Unit	Description
$supply\_Mt_{i,c,t}$	Mt	production of coal type *c* at node *i* and year *t*
$transp\_Mt_{i,j,c,t}$	Mt	transport of coal type *c* between nodes *i* and *j* in year *t*

Table 3. Model sets in the IL-CCM

Set	Description	Notation
(i,j)	Set of all nodes	$\in I$
p	Subset of nodes that are ports	$\in P \subset I$
s	Subset of nodes that are steel plants	$\in S \subset I$
(k,k')	Index of node types	$\in K$ = [Mine, basin, railway stop, city center, port, navigation waypoint (river), navigation waypoint (ocean), power plant, power plant unit, steel plant, provincial power demand center, provincial steel demand center]
t	Years	$\in T$ = [2015,…,2030]
c	Coal types	$\in C$ = [Thermal coal, HCC, SCC, PCI]
v	Bins with average calorific value of coal types	$\in V$ = [0, 2250, 2500,…,7000]
m	Transportation modes	$\in M$ = [rail, truck, river barge, ocean-going ship]

Table 4. Model parameters in the IL-CCM

Parameter	Unit	Description
$elec\_demand_{i,t}$	PJ	demand for coal-fired power generation in node *i* and year *t*
$other\_demand_{i,t}$	PJ	demand for thermal coal for other uses in node *i* and year *t*
$steel\_demand_{i,t}$	Mt	demand for steel in node *i* and year *t*
CV_v	kcal/kg	calorific value of thermal coal in bin *v*
kcal_to_PJ	-	conversion factor for energy content, from kcal/kg to PJ/Mt
HCC_c	-	dummy variable indicating coal type is hard coking coal
SCC_c	-	dummy variable indicating coal type is semi-soft coking coal
PCI_c	-	dummy variable indicating coal type is pulverized coal for injection
$prod\_cost_{i,c,t}$	$/t	production cost of coal type *c* at node *i* and year *t*
$prod\_capa_{i,c,t}$	Mt	production capacity of coal type *c* at node *i* and year *t*
$transp\_capa_{i,j,t}$	Mt	transport capacity between nodes *i* and *j* in year *t*
$port\_capa_{p,t}$	Mt	handling capacity of port *p* in year *t*
$elec\_capa_{i,j,t}$	PJ	electrical transmission capacity between between nodes *i* and *j* in year *t*
$stpt\_capa_{s,j,t}$	Mt	production capacity of steel plant *s* in year *t*
$conv\_eff_{i,j}$	-	efficiency of electrical conversion or transmission between nodes *i* and *j*
$distance_{i,j}$	km	distance between nodes *i* and *j*
$transp\_cost_{m,t}$	$t·km	variable cost for a transport mode *m* in year *t*
$handling\_cost_{k,k',t}$	$/t	fixed handling charge for transport between node of type *k* and type *k'* in year *t*
$transm\_cost_{i,j,t}$	$/PJ	variable cost for a transmission of electricity between nodes *i* and *j* in year *t*
